# Systemic and locoregional therapies for cutaneous metastases from breast cancer: state of art and new frontiers in treatment approach

**DOI:** 10.1177/17588359251387539

**Published:** 2025-12-05

**Authors:** Francesco Russano, Davide Brugnolo, Michele Bottosso, Luigi Dall’Olmo, Paolo Del Fiore, Rino Baldan, Massimo Ferrucci, Noemi Musilli, Maria Vittoria Dieci, Valentina Guarneri, Marco Rastrelli, Simone Mocellin

**Affiliations:** Soft-Tissue, Peritoneum and Melanoma Surgical Oncology Unit, Veneto Institute of Oncology IRCCS, Padova, Italy; Department of Surgery, Oncology and Gastroenterology (DISCOG), University of Padua, Padua, Italy; Department of Surgery, Oncology and Gastroenterology (DISCOG), University of Padua, Padua, Italy; Division of Oncology 2, Istituto Oncologico Veneto IRCCS, Padova, Italy; Soft-Tissue, Peritoneum and Melanoma Surgical Oncology Unit, Veneto Institute of Oncology IRCCS, Padova, Italy; Department of Surgery, Oncology and Gastroenterology (DISCOG), University of Padua, Padua, Italy; Soft-Tissue, Peritoneum and Melanoma Surgical Oncology Unit, Veneto Institute of Oncology IRCCS, Padova, Italy; Department of Surgery, Oncology and Gastroenterology (DISCOG), University of Padua, Padua, Italy; Breast Surgical Unit, Veneto Institute of Oncology IRCCS, Via Gattamelata, 64, Padova 35121, Italy; Breast Surgical Unit, Veneto Institute of Oncology IRCCS, Padova, Italy; Department of Surgery, Oncology and Gastroenterology (DISCOG), University of Padua, Padua, Italy; Division of Oncology 2, Istituto Oncologico Veneto IRCCS, Padova, Italy; Department of Surgery, Oncology and Gastroenterology (DISCOG), University of Padua, Padua, Italy; Division of Oncology 2, Istituto Oncologico Veneto IRCCS, Padova, Italy; Soft-Tissue, Peritoneum and Melanoma Surgical Oncology Unit, Veneto Institute of Oncology IRCCS, Padova, Italy; Department of Surgery, Oncology and Gastroenterology (DISCOG), University of Padua, Padua, Italy; Soft-Tissue, Peritoneum and Melanoma Surgical Oncology Unit, Veneto Institute of Oncology IRCCS, Padova, Italy; Department of Surgery, Oncology and Gastroenterology (DISCOG), University of Padua, Padua, Italy

**Keywords:** breast cancer, electrochemotherapy, locoregional therapy, skin metastases, systemic therapy

## Abstract

Cutaneous metastases from breast cancer, although relatively uncommon, represent the most frequent form of skin metastases overall and pose a significant clinical and therapeutic challenge. Their presence classifies the disease as stage IV, typically prompting the initiation or modification of systemic treatment. However, current clinical guidelines do not distinguish between cutaneous and visceral metastases, which may lead to unnecessary alterations in systemic therapy—even when visceral disease remains well controlled—potentially compromising an otherwise effective regimen. This review provides a comprehensive overview of systemic and loco-regional treatment options for cutaneous breast cancer metastases, including current guidelines stratified by tumor subtype. Special attention is given to loco-regional therapies such as electrochemotherapy, radiotherapy, surgical excision, photodynamic therapy, intralesional agents, and topical treatments, all of which can be integrated with systemic therapy to improve local disease control, reduce symptoms, and enhance patient quality of life. We propose an integrated and personalized therapeutic model that combines systemic and loco-regional approaches, supported by a decision-making flowchart designed to assist clinicians in optimizing treatment strategies. By adopting a multidisciplinary perspective, this approach aims to improve both local and systemic disease management, clinical outcomes, and patient well-being. Further research is warranted to refine therapeutic combinations, establish standardized protocols, and fully realize the clinical benefits for patients with metastatic breast cancer presenting with cutaneous involvement.

## Introduction

Cutaneous metastases involve the spread of malignant cells from a primary tumor to the skin. They represent rare clinical entities, with an overall incidence ranging from 0.6% to 10.4% among cancer patients, accounting for approximately 2% of all skin cancers. Metastatic spread may occur through the bloodstream, lymphatic vessels, direct invasion, or dissemination following surgery.^1^ Cutaneous metastases typically manifest months or years after the diagnosis of the primary tumor^2^; however, it is estimated that in about one-third of cases cutaneous metastasis can be the initial presentation of the tumor.

The appearance of cutaneous metastases can be the first manifestation of disease recurrence, and it is estimated that concomitant visceral metastases are present in 79% of cases.^3^ Advances in metastatic cancer treatment have prolonged patient survival but have also increased the risk of complications associated with advanced disease, such as cutaneous metastases. These metastases represent a significant source of morbidity, as they substantially impair patient’s quality of life. They can serve as sites for infection, bleeding, disfigurement, and pain.^4^ Several studies highlight that patients often perceive cutaneous metastases as the complication with the greatest impact on their quality of life.^5^

Several cancer types can metastasize the skin. A 2019 retrospective study of 164 patients reported breast cancer had the highest incidence of cutaneous metastases,^6^ consistent with findings from a 2003 meta-analysis.^7^ The distribution of cutaneous metastases generally mirrors the sex-specific incidence of primary tumors: in women, breast cancer is the predominant source, accounting for up to 70% of cases. On the other hand, in men, lung cancer is the most frequent origin, whereas breast cancer, although rare, is also showing a rising incidence worldwide.^8,9^ The high proportion of breast cancer-related cases may be explained by its high prevalence and by the potential for contiguous spread, given the breast’s superficial location.

A multimodal approach is now considered the preferred treatment strategy. This review comprehensively examines the effectiveness of systemic and locoregional treatments for cutaneous metastases from breast cancer, with a particular focus on the integration of systemic therapies with locoregional approaches in patients with good visceral control but inadequate response at the cutaneous level.

## Epidemiology

Breast cancer is among the most common malignancies in women and represents the second cause of cancer-related death among women. Despite its high incidence, mortality rates have decreased in developed countries due to early diagnosis and advancements in treatment for both early and advanced stages of disease.^10,11^ However, approximately 10% of breast cancer patients develop distant metastases and metastatic breast cancer is generally considered an incurable disease.^12,13^ Breast cancer most commonly metastasizes to lungs, bones, and brain,^14^ but cutaneous metastases also occur in about 23.9% of patients with advanced disease. Cutaneous metastases mostly involve chest and abdomen, though they can also appear on limbs and on the head and neck regions.^15,16^ These metastases typically spread through lymphatic, hematogenous, or contiguous routes.^17^ Cutaneous metastases are generally a secondary manifestation of advanced breast cancer but may also be the first sign of the disease. Most of these metastases arise from adenocarcinomas and are commonly localized to the anterior chest, facilitated by both contiguity and regional lymphatic drainage.^18^

It has been noted that patients with only cutaneous metastases generally have a better prognosis compared to those with stage IV breast cancer involving visceral metastases.^19^ Nonetheless, the prognosis is poorly correlated with the number of cutaneous lesions. Mortality is estimated to be around 70% within the first year of diagnosis, and no curative treatment is currently available. Management focuses on palliative care to control symptoms, improve quality of life, and slow disease progression.

## Clinical classification of cutaneous metastasis

The clinical presentation of cutaneous metastases is highly variable, making differential diagnosis from other skin neoplasms often challenging. These metastases may appear as normochromic papules or nodules, but distinct forms also exist, including telangiectatic carcinoma, erysipeloid carcinoma, cuirass carcinoma, and neoplastic alopecia^20^:

1. Telangiectatic carcinoma: characterized by purple papules on a telangiectatic surface, often located near the scar of a previous surgery. This form of metastasis results from the dissemination of malignant cells to the skin via the bloodstream, with an estimated incidence ranging from 8% to 11% of cases.
2. Erysipeloid carcinoma (or inflammatory metastatic carcinoma): a rare form of cutaneous metastasis, manifests as a well-defined erythematous lesion resembling an infection, such as erysipelas. This type of metastasis spreads rapidly both locally and systemically and is associated with a poor prognosis. The dissemination primarily occurs through lymphatic vessels,^21^ with an estimated incidence ranging from 3% to 6%.^22^
3. Cuirass carcinoma: a very rare cutaneous presentation, confined to the chest wall. It is characterized by the formation of hard, infiltrative skin plaques, resembling scleroderma, with an incidence of 3%–4%.
4. Neoplastic alopecia: presents as hard nodules or plaques on the scalp, associated with alopecia and destruction of hair follicles. Metastatic spread occurs via the hematogenous route, with a prevalence ranging from 2% to 12%.
5. Zosteriform papules: These are lesions that appear in a distribution similar to herpes zoster, but their presence is less common in breast cancer metastases.

In 80% of cases, cutaneous metastases from breast cancer present as nodular lesions, typically measuring between 1 and 3 cm. These lesions may appear as solitary or multiple nodules, located within the dermis or subcutaneous tissue. Their color often resembles that of healthy skin but can also range from red-brown to pink or black. In some instances, the lesions may ulcerate and become secondarily infected.^23^

## Pathological anatomy

From a pathological anatomy perspective, cutaneous metastases exhibit distinct and characteristic patterns, which vary depending on the clinical manifestation. Nodular lesions are marked by neoplastic cells arranged in nests and cords, surrounded by fibrosis within collagen bundles in the dermis. In the telangiectatic pattern, aggregates of neoplastic cells and atypical erythrocytes are observed within dilated vessels in the papillary and/or reticular dermis. The erysipeloid pattern is characterized by metastatic tumor cells densely packed within dilated superficial and deep lymphatic vessels, often accompanied by a perivascular infiltrate of lymphocytes and plasma cells. En-cuirasse carcinoma develops through dense fibrosis with few neoplastic cells, sometimes arranged in a characteristic single-file pattern between collagen bundles in the dermis. Lastly, neoplastic alopecia is defined by the presence of small tumor cells arranged in cords, with individual cells destroying hair follicles and inducing fibroplasia.^24^ The “Non-special Type” histotype is the most frequent cause of cutaneous metastases, followed by invasive lobular carcinoma, with other histotypes occurring less frequently.^25^ Additionally, several studies have shown that the frequency of cutaneous metastases varies across different breast cancer subtypes, with a particularly high incidence observed in triple-negative tumors.^26^

From a molecular point of view, metastases are associated with the acquisition of additional mutations compared to the primary carcinoma.^27^ The types of mutations acquired during the metastatic process depend on both the molecular subtype of the primary tumor and the metastatic site. In a study by Rinaldi et al., which included 118 cases of cutaneous metastases from breast cancer, it was found that mutations in the NOTCH1 gene are specific to cutaneous metastases, while a group of other mutations was observed in only a limited number of cases.^28,29^

## Diagnosis

An accurate diagnosis of cutaneous metastases requires a thorough clinical examination, imaging tests, and histopathological and immunohistochemical analyses. The histopathological and immunohistochemical characteristics of cutaneous metastases tend to be similar to those of the primary tumor, although neoplastic cells may be less differentiated.^30^

An essential aspect of the differential diagnosis of primary tumors is the immunohistochemical pattern. Cutaneous metastases from breast cancer typically express CK7, CK19, estrogen and progesterone receptors, mammaglobulin, carcinoembryonic antigens, and E-cadherin. They are negative for CK20, CK5/6, CD10, and TTF-1. Human epidermal growth factor receptor 2 (HER2) is amplified in approximately 20%–30% of invasive breast carcinomas.^31^

For cutaneous metastases from breast cancer, the immunohistochemical evaluation should focus on the expression of the following markers typical of breast cancer:

- Mammaglobin and GCDFP: Mammaglobin is the most sensitive marker for breast cancer, while GCDFP (gross cystic disease fluid protein) is the most specific. These markers are expressed in most luminal and HER2-positive tumors but are less sensitive in triple-negative tumors (35% and 16%, respectively). Both markers are also expressed in cutaneous metastases of adnexal origin.
- GATA3: GATA3 is a transcription factor involved in the differentiation of various tissues, including the mammary luminal epithelium. However, it is also expressed in urothelial, trophoblastic, salivary, and pancreatic tumors.
- SOX10: SOX10 is a transcription factor involved in the differentiation and survival of neural crest cells. SOX10 has been found positive in 66%–74% of triple-negative and metaplastic tumors but only in 5% of non-triple-negative tumors.^32^

Table 1 summarizes the expression of immunohistochemical markers in breast cancer.

**Table 1.** Immunohistochemical markers for cutaneous metastases from breast cancer.

Origin	Histopathology	Immunohistochemical markers	
		Positive	Negative
Breast	Ductal	CK7, estrogen-R, progesteron-R, GCDFP-15, CEA, c-erbB-2, mammaglobin, E-cadherin	CK20, CK5/6
	Lobular carcinoma	CK7, estrogen-R, progesteron-R, GCDFP-15, CEA, EMA, mammaglobin	S100, E-cadherin, podoplanin, P63
	Inflammatory carcinoma	CD31, podoplanin	Podoplanin
	Teleangiectatic carcinoma	CD31	Podoplanin
	Mammary Paget disease	MUC1, CK7	MUC2, MUC5AC, CK20

CEA, carcinoembryonic antigen; EMA, epithelial membrane antigen; GCDFP, gross cystic disease fluid protein.

In case of poorly differentiated tumors or triple-negative neoplasms, it may be difficult to establish a clear origin, and the tumor might be simply classified as adenocarcinoma, squamous cell carcinoma, or undifferentiated carcinoma. In such situations, the tumor is referred to as “carcinoma of undefined nature.” Therefore, it will be necessary to complement the diagnosis with further investigations, such as CT (computed tomography) and/or MRI (magnetic resonance imaging), and mammography if a mammary origin is suspected.

## Treatment

### Systemic therapies for cutaneous metastases from breast cancer

Currently, there are no specific guidelines for the systemic treatment of cutaneous metastases from breast cancer, and therapeutic strategies should follow the recommendations for advanced breast cancer.^33^ Treatment algorithm for advanced breast cancer is primarily based on the disease subtype according to hormone receptor (HR) and HER2 status, and should take into account disease burden, prior therapies, patient performance status and comorbidities. Notably, in breast cancer, biopsy at recurrence and throughout the course of the disease is crucial for recharacterizing receptor status, as biological changes may occur over time.^34,35^ In this context, skin represents a particularly accessible site, making it advantageous for biopsies. For instance, a high discordance rate of HER2-expression was reported in soft tissue/skin samples (39.8%) compared to primary breast cancer,^36^ while a systematic review evaluated PD-L1 positivity according to metastatic site reported a positivity rate of 39% in skin samples.^37^ With the continuous evolution of treatment, algorithms and the incorporation of new targeted agents in the era of precision medicine,^38,39^ cutaneous metastases represent a valuable opportunity for disease characterization and for promptly monitoring treatment response.

**Figure 1.**
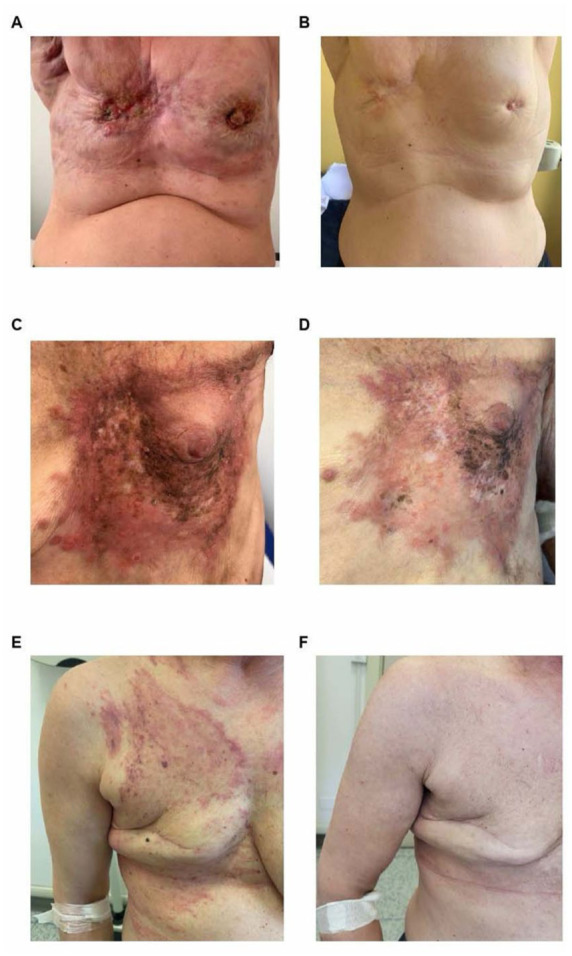
Patient with cutaneous metastases from a hormone receptor-positive/HER2-negative breast cancer at baseline (a) and after 58 cycles of letrozole plus abemaciclib (b). Patient with cutaneous metastases for hormone receptor-positive/HER2- low breast cancer at baseline (c) and after 11 cycles of trastuzumab deruxtecan (d). Patient with cutaneous metastases for hormone receptor-negative/HER2-positive breast cancer at baseline (e) and after 20 cycles of trastuzumab deruxtecan (f).

For patients with HR+/HER2-metastatic breast cancer, endocrine therapy in association with a CDK4/6 inhibitor is the cornerstone of treatment.^40–42^ Several case reports have reported major responses from CDK4/6 inhibitors in the case of skin lesions, confirming their efficacy in this setting (Figure 1(a) and (b)).^43,44^ In the second line, based on the agents previously used, determination of estrogen receptor 1 (ESR1) mutations and PI3K-pathway alterations may allow access to further endocrine-based strategies, that is, the oral selective estrogen receptor degrader (SERD) elacestrant and fulvestrant-capivasertib combination.^45,46^ In later lines, besides single-agent chemotherapy, antibody drug-conjugates (ADCs) have recently entered the treatment algorithm of HR+ disease, with trastuzumab deruxtecan being approved in the HER2-low disease and sacituzumab govitecan in pretreated patients (Figure 1(c) and (d)).^47,48^

Anti-HER2-targeted therapies represent the cornerstone of treatment for metastatic HER2+ breast cancer. The first-line standard of care includes trastuzumab and pertuzumab in combination with a taxane,^49^ while in the second-line setting, trastuzumab deruxtecan has emerged as the preferred option.^50^ Subsequent treatment lines include tucatinib combined with trastuzumab and capecitabine, T-DM1, as well as various combinations of trastuzumab and chemotherapy. Case reports have described remarkable responses to ADCs and tyrosine kinase inhibitors in skin metastases from HER2+ breast cancer, confirming the effectiveness^51^ of anti-HER2 therapies on this specific site (Figure 1(e) and (f)).^52,53^

Triple-negative breast cancer represents the most aggressive breast cancer subtype, and for most cases, systemic therapy generally requires chemotherapy-based regimens, with the addition of immune checkpoint inhibitors in case of PD-L1-positive tumors.^54,55^ For patients harboring germline BRCA1/2 pathogenic variants, Poly ADP-ribose Polymerase (PARP) inhibitors, such as olaparib or talazoparib, represent effective therapeutic options.^56,57^ In later lines, sacituzumab govitecan, an antibody-drug conjugate targeting Trop-2, has shown substantial clinical benefit also in patients with skin metastases,^58^ while trastuzumab deruxtecan has been approved in pre-treated HER2-low breast cancers.^58,59^

### Local-regional treatment

The treatment of cutaneous metastases is typically multimodal, with systemic therapy complemented by local-regional therapies to achieve better local responses.

Local-regional therapies can play an adjunctive role in controlling local disease. A 2014 meta-analysis of 47 prospective studies on cutaneous metastases reported a complete response rate of 35.5% and an objective response rate of 60.2%, with a recurrence rate of 9.2% and grade 3 toxicity observed in less than 6% of patients. Notably, in cases of breast cancer metastases, which comprised 13.5% of the reviewed cases, the objective response rate was 54%.^60^ These treatments are characterized by a low incidence of grade 3 toxicity, making them well-suited for integration with systemic therapies. Importantly, local-regional treatments can often be administered alongside systemic therapy, without requiring interruption or delay of the latter.

The techniques employed for local-regional treatment of cutaneous metastases include:

— *Electrochemotherapy*: Electrochemotherapy (ECT) combines the administration of a cytotoxic agent, delivered either intravenously or directly into the tumor (intratumorally (IT)), with the application of short, high-voltage electric pulses to the tumor area. These pulses transiently increase the permeability of tumor cell membranes, thereby enhancing intracellular drug uptake and potentiating cytotoxic effects.^61,62^ Agents most commonly used include bleomycin and cisplatin. While bleomycin can be delivered both IT or systemically, cisplatin is generally injected directly IT to achieve optimal therapeutic outcomes.^63^ When drugs are given systemically, the electric pulses must be applied at the time of peak plasma concentration, typically between 8 and 28 min after injection. In contrast, for intratumoral drug delivery, pulses are usually administered within 1–10 min following injection.^64^ The electric pulses temporarily disrupt the lipid bilayer of tumor cell membranes, allowing otherwise poorly permeant drugs, such as bleomycin or cisplatin, to enter the cells more efficiently.^65^ Once inside, bleomycin exerts its effect primarily by causing DNA strand breaks. If these lesions remain unrepaired, dividing cells undergo chromosome fragmentation and eventually die, whereas quiescent, non-dividing cells are relatively spared.^66,67^ ECT also provokes transitory local ischemia and permanent vascular damage, contributing to reducing tumor perfusion.^68,69^ Moreover, by promoting tumor antigen release, ECT may trigger a systemic immune response that is selective for neoplastic cells. Systemic immunity is induced and can be upregulated by additional treatment with biological response modifiers.^70^ ECT is generally well tolerated. The most frequently reported adverse events are erythema, edema, ulceration, and persistent skin pigmentation. Systemic toxicity is rare; however, severe complications such as respiratory failure have been reported in patients with significant comorbidities. Clinical evidence strongly supports ECT efficacy. A 2021 systematic review and meta-analysis involving 1161 patients with metastatic cutaneous melanoma demonstrated 1-year local control rates between 54% and 89%, with overall survival ranging from 67% to 89%. In a pooled analysis of 16 studies including 440 patients, complete response rates varied between 33% and 75.3%, while partial responses ranged from 15% to 67%.^71^ Finally, a meta-analysis of 55 studies published from 1993 to 2021 on ECT for cutaneous metastases of various origins reported an average complete response rate of 81.5%, with no significant differences observed across different histopathological tumor subtypes.^72,73^ It should be emphasized that the majority of evidence supporting the efficacy of ECT comes from studies in melanoma and other primary skin cancers. Data specifically addressing cutaneous metastases from breast cancer, however, remain limited.
— *Photodynamic therapy*: Photodynamic therapy (PDT) is a local-regional treatment that uses a photosensitizing agent, such as methyl aminolevulinate (a porphyrin), and a light beam to generate reactive oxygen species (ROS) at the cellular level, with cytotoxic effects on tumor cells.^74^ The photosensitizing agent accumulates in tumor cells, and when exposed to light of a specific wavelength, it triggers the production of ROS, which damages metastatic cells. The choice of wavelength depends on the type of lesion and the photosensitizer used.^75^ Blue light (around 400 nm) is preferred for superficial skin lesions, while red light (around 600 nm) is used for larger tumors that require deeper penetration. PDT is a selective, minimally invasive, and outpatient procedure with minimal side effects, such as redness, swelling, or localized pain. However, it is mainly indicated in selected cases, when other local-regional treatments are not applicable. Recent studies are evaluating the combination of PDT with immunotherapy, with promising results. Moreover, PDT combined with nanoparticles for targeted delivery of immunotherapeutic drugs is being investigated to enhance its effectiveness.^76^ A meta-analysis conducted on nine studies involving 102 patients reported a response rate ranging from 11.1% to 92%, with a follow-up period of 6–12 months. PDT has proven particularly useful for small lesions, while it is less effective for larger and more voluminous metastases.
— *Radiotherapy*: Radiotherapy employs ionizing radiation, delivered by a linear accelerator, to damage tumor cell DNA through the generation of free radicals, ultimately inducing cell death. Among locoregional treatment modalities, external-beam radiotherapy (EBRT) currently represents the most established and widely used palliative option for patients with symptomatic cutaneous or chest wall involvement from breast cancer. It provides effective local disease control by reducing tumor burden while offering additional advantages, including non-invasiveness, broad accessibility, and efficacy at any tissue depth.^77^ For superficial chest-wall or skin disease, both photons (often with a daily bolus) and electrons can be employed. EBRT reliably relieves pain, bleeding, infections, ulcerations, and foul-smelling wound discharge. These conditions, common in patients with advanced ulcerating or fungating breast lesions, cause significant physical and social discomfort, and their control substantially improves quality of life, even in the absence of curative intent. In hemorrhagic or heavily bleeding fungating tumors, EBRT also achieves prompt and effective hemostasis.^78^ Although data specifically focused on cutaneous metastases are limited, robust evidence from locoregionally advanced breast cancer—where skin involvement is frequent—supports the palliative role of radiotherapy.

A recent large multi-institutional study by Jacomina et al. analyzed 164 patients with symptomatic, inoperable, locoregionally advanced breast cancer treated with palliative EBRT. The study reported excellent symptomatic responses and durable local control across different fractionation regimens, with pain relief, bleeding reduction, and improved wound healing, all with acceptable toxicity. Symptom improvement correlated with radiation dose, whereas overall survival remained poor, highlighting the central role of palliation in minimizing symptom burden while maximizing quality of life.^79^

Palliative RT can be delivered with different fractionation schedules, tailored to patient tolerance and tumor response. Prospective studies, including the HYPORT and HYPORT-B phase I/II trials, have confirmed the feasibility and safety of hypofractionated and ultrahypofractionated regimens. These include 26 Gy in 5 fractions, “quad-shot” schedules (≈14–14.8 Gy in 4 fractions over 2 days, repeatable if needed), 30 Gy in 10 fractions, or even a single 8-Gy fraction. Such regimens are well tolerated, shorten treatment duration, provide durable palliation with rapid symptom relief—including partial or complete healing of lesions—and minimize toxicity and interruptions to systemic therapy.^80^ For these reasons, they are increasingly considered a standard for locoregional symptom control, particularly when systemic therapies are ineffective or discontinued due to toxicity.^81,82^

Regimen selection should balance durability (favoring 26–30 Gy or higher) with treatment convenience and integration with systemic therapy (favoring short or single-fraction schedules). Fractionated regimens often yield more sustained responses than single 8-Gy fractions, although the latter remain invaluable for immediate symptom control, rapid hemostasis, or in frail patients, minimizing treatment burden and systemic therapy interruption.^83,84^ In selected cases requiring more durable control, such as large metastases or recurrences in the chest wall, higher doses up to 50 Gy in 25 fractions may be delivered.

When planning palliative radiotherapy, previous irradiation to the breast or chest wall must be carefully considered, as re-irradiation carries increased risk of severe toxicity, including skin ulceration, impaired wound healing, and necrosis. Nevertheless, re-irradiation is feasible and increasingly applied in selected patients when symptoms warrant, prior dosing and patient goals permit. With modern techniques and adequate treatment intervals, in conjunction with novel devices designed to facilitate complex wound healing, acceptable toxicity rates can be achieved.^85–87^

Beyond symptom control, radiotherapy can synergize systemic therapies, either sequentially or concurrently, with an acceptable toxicity profile and potential immunogenic effects. Indeed, it has been shown to induce immunogenic cell death and enhance systemic anti-tumor immune responses, although without a demonstrated survival benefit to date. While ECT remains an effective option—especially for small superficial nodules—its limited availability, anesthesia requirement, and suitability only for low-volume disease make EBRT the more established, accessible, and easily deliverable locoregional palliative option for symptomatic cutaneous and chest wall metastases.^88^

In summary, EBRT represents a cornerstone of locoregional palliation in advanced breast cancer. Its proven efficacy, accessibility, and favorable toxicity profile make it a key component of multidisciplinary care, complementing systemic therapies and providing substantial improvements in quality of life for patients with advanced disease.

— *Brachytherapy*: It involves the direct application of a radioactive source within or near the tumor,^89^ and it is less commonly used for the treatment of cutaneous metastases.^90^ However, one study showed that radiotherapy achieved local control in 89% of patients, with a median follow-up of 16 months.^91^
— *Intralesional therapy*: Intralesional therapy (ILT) involves the direct injection of anticancer drugs or therapeutic agents into the metastatic area, allowing for high local drug concentrations with minimal systemic effects. Commonly used drugs include doxorubicin and paclitaxel, as well as experimental agents such as tyrosine kinase inhibitors (e.g., erlotinib). In addition, immunotherapeutic agents that can be injected to stimulate a local immune response include IL-2 (interleukin 2), therapeutic vaccines such as Bacillus Calmette-Guérin (BCG), and granulocyte-macrophage colony-stimulating factor (GM-CSF). ILT is a quick, effective treatment with a low incidence of side effects, often performed under local anesthesia. It is particularly useful for patients with limited metastatic disease or those who are not candidates for systemic therapy. The use of intralesional agents such as IL-2, BCG, or GM-CSF has been mainly explored in melanoma. Data regarding breast cancer cutaneous metastases are poor and not conclusive.
1. BCG: Bacillus Calmette-Guérin is an attenuated vaccine of *Mycobacterium bovis* that stimulates a strong local immune response, promoting the activation of T lymphocytes against tumor cells. Although BCG has shown some benefit in managing cutaneous metastases, its side effects and lower efficacy compared to other local options limit its use.^92^
2. IL-2: This cytokine stimulates a specific immune response against tumor cells by activating helper T cells and enhancing the inflammatory response.^93,94^
3. GM-CSF: This cytokine^95,96^ stimulates the differentiation of myeloid cells and promotes immune surveillance, with evidence suggesting potential benefit in activating a local immune response against cutaneous metastases.^97,98^
— *Topical therapy*: Topical treatments can be used for thin, superficial cutaneous metastases. Locally applied antineoplastic agents include 5-fluorouracil cream, miltefosine, 5% methotrexate (administered with oxygen flow to facilitate absorption), and imiquimod cream, which stimulates immune response against tumor cells. These treatments are generally well tolerated and show good responses when combined with other local-regional therapies.^99^ In cases of ulceration or superimposed infection, medicated dressings with antibacterials (such as silver sulfadiazine) and hydrocolloid dressings can be used.
1. OFAMTX (5% Methotrexate with oxygen flow assistance)^100^: This treatment is particularly effective for extramammary Paget’s disease, with bi-weekly sessions for 2 weeks. Topical administration significantly reduces systemic side effects compared to oral methotrexate.^101^
2. Paclitaxel: Topical administration of submicronic paclitaxel has shown good penetration through the epidermis and dermis, with a low incidence of systemic side effects.^102^
3. Imiquimod: A topical agonist of toll-like receptor 7, which stimulates the production of immune cytokines. It has been shown that the combination of 5% imiquimod with nab-paclitaxel is locally effective, with a low incidence of side effects.^103^ In a study of 13 patients, a clinical response was seen in 50.5% of the lesions, with 40.7% of these being a complete response.^99^
4. Miltefosine: An antitumor drug^104^ that destabilizes the membranes of tumor cells, leading to apoptosis. It is used in the topical treatment of cutaneous metastases from breast cancer.
— *Laser treatments*: Laser treatments, particularly CO_2_ laser, can be used palliatively for the treatment of cutaneous metastases from breast cancer. The CO_2_ laser is one of the most common tools in dermatology and oncology for treating various skin conditions, including cutaneous metastases. The CO_2_ laser emits a focused, high-energy laser light that enables the ablation of tumor tissue. It is particularly effective due to its ability to remove layers of tissue with precision, minimizing damage to surrounding healthy tissue. This is a precise system (it allows targeted treatment of tumor lesions, minimizing damage to the surrounding healthy tissue), equipped with photocoagulation, thus reducing the risk of bleeding during and after the procedure. This treatment also allows for a rapid recovery. However, its effectiveness can vary depending on the nature and depth of the cutaneous metastases.^105^ Therefore, laser is considered a palliative treatment rather than a curative one. A thorough pre-operative evaluation is required to determine whether it is the most appropriate treatment. Despite these limitations, the CO_2_ laser has proven effective in treating cutaneous metastases that are not eligible for other treatments, such as surgery. The laser works by evaporating the water contained in the cells, inducing cell death directly in tumor cells with minimal damage to the surrounding healthy tissue. Although laser treatment is currently mainly indicated for cutaneous metastases that are not suitable for surgical resection or other loco-regional treatments, the results obtained in the treatment of in-transit metastases from melanoma suggest its potential for broader future use, also due to the negligible side effects. However, no prospective studies have specifically assessed this approach in breast cancer.
— *Surgery*: Surgical removal of cutaneous metastases is considered when the metastases cause symptoms such as pain or discomfort, or for aesthetic reasons. Surgery can be part of a multimodal approach, combined with systemic treatments. Surgical techniques vary depending on the size and location of the metastases. The procedure may involve simple resection or, in some cases, more advanced techniques, such as laser surgery or PDT. However, surgical excision of cutaneous metastases without the integration of other loco-regional or systemic treatments has shown poor results in both local and distant metastasis control and is associated with a high risk of local recurrence. A study by Salvadori et al. analyzed 39 patients with breast cancer and cutaneous metastases treated with wide excision of skin lesions. Among these, 26 patients received adjuvant chemotherapy, and the results showed a median follow-up of 48 months, with local disease control maintained in 32 patients, 21 of whom were disease-free at the end of the follow-up.^106^ Unlike primary skin tumors, there are no standardized guidelines for the width of resection margins in cutaneous metastases. Therefore, surgical resection is generally considered only suitable for cutaneous metastases localized in small areas that can be easily closed or reconstructed using techniques such as local skin advancement or skin flaps.

## Discussion

Cutaneous metastases from breast cancer are a distant disease localization (therefore classified as stage IV) and should be distinct from the direct local invasion of the skin and chest wall by primary breast carcinoma. Cutaneous metastases represent a complex therapeutic challenge and are expected to become increasingly prevalent in the future, driven by advancements in disease control and prolonged survival of patients with stage IV breast cancer, thanks to emerging therapeutic options.

According to all international guidelines, the treatment of locally advanced or metastatic breast cancer is primarily based on systemic therapy. This concept also applies to patients with cutaneous disease, where the conventional approach, usually following a biopsy-confirmed diagnosis of metastatic cutaneous breast cancer, is to initiate systemic treatment. Although most studies were not designed for this specific scenario, this strategy is based on the premise that cutaneous metastases, like other metastatic lesions, indicate a disseminated disease and therefore a stage IV carcinoma, for which systemic therapy is recommended. Several experiences and case series have reported remarkable responses of skin lesions to various classes of systemic agents, supporting their use in this disease localization as well.

In addition to systemic treatments, numerous studies have demonstrated the effectiveness of loco-regional therapies in controlling cutaneous metastases from various origins. These treatments have shown excellent local regression rates and generally exhibit a very low toxicity profile. The favorable safety profile enables their seamless integration with systemic therapies without requiring prolonged interruption of systemic agents, which remain essential for controlling distant disease. At the same time, these treatments effectively contribute to local disease control and enhance patients’ quality of life.

**Figure 2.**
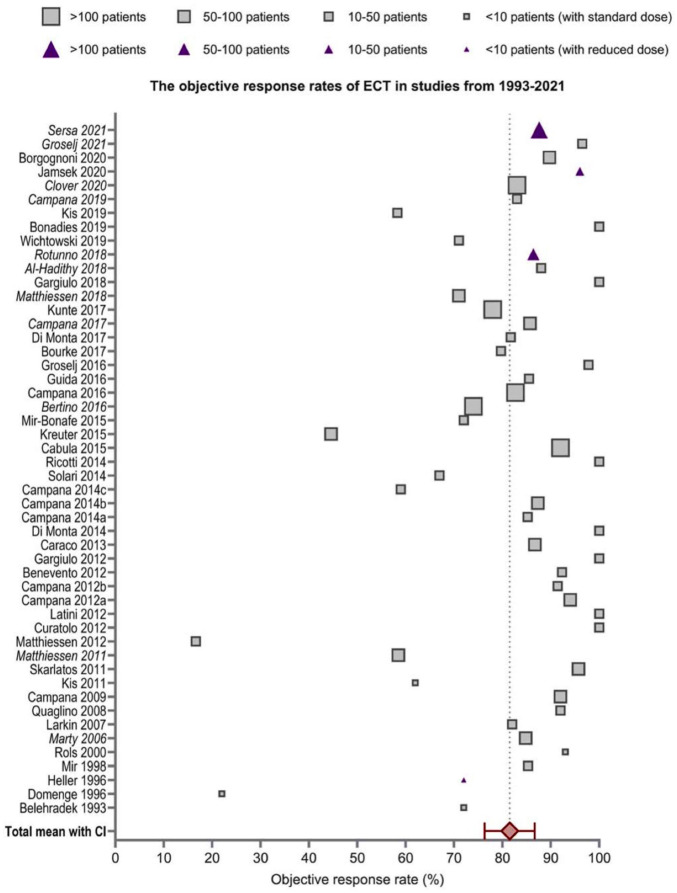
Objective response rates of ECT reported in the 55 studies,^108^ including the pooled mean with confidence intervals. Squares indicate studies with a standard dose of bleomycin and triangles indicate studies with a reduced dose of bleomycin. The size of the square/triangle is dependent on the study size. Studies written in italics are from the InspECT network. ECT, electrochemotherapy.

**Figure 3.**
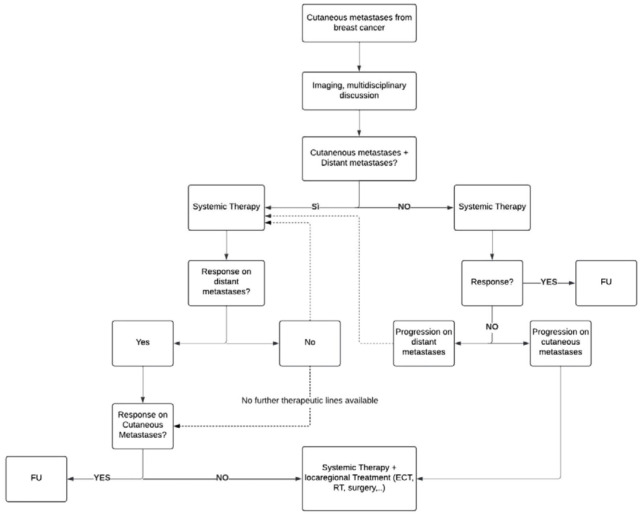
Flowchart for the treatment of cutaneous metastases from breast cancer. ECT, electrochemotherapy; FU, follow up; RT, radiotherapy.

In a 2022 systematic review,^107^ the authors analyzed the SCOPUS and MEDLINE databases, identifying 110 articles on the management of cutaneous metastases from breast cancer. Most studies focused on chemotherapy,^42^ followed by ECT,^16^ radiotherapy,^15^ aromatase inhibitors,^13^ surgery,^12^ and topical therapies.^10^ Notably, the review examined the response rates associated with various loco-regional treatments (Figure 2). The most commonly used approaches, which were ECT, radiotherapy, and surgical excision (when combined with systemic therapy), demonstrated favorable response rates (Figure 3). The choice of the most appropriate loco-regional treatment depends on several factors, including the number and size of the lesions:

- Surgery: Indicated for a single resectable nodule with clear margins, provided that complex reconstructions are not required, as these could delay the resumption of systemic therapy.
- Electrochemotherapy: recommended for nodules smaller than 3 cm, as it has demonstrated the highest complete response rate, particularly for intralesional treatments. This approach is associated with a very low incidence of major side effects, allowing for compatibility with systemic therapies without requiring treatment interruption.
- Radiotherapy: used for lesions larger than 3 cm or in cases of disseminated disease, radiotherapy is a well-established technique with strong efficacy.
- Topical therapies and PDT: These treatments are rarely used for cutaneous metastases from breast cancer due to their limited effectiveness (Table 2).

**Table 2.** Selected study outcomes from non-surgical treatment modalities.

Modality	Author	Year of publication	Treatment	No. of patients (*n*)	Longest follow-up	Response rate	Response rate (*n*)
ECT	Falk	2018	ECT	6	12 Months	CR: 68% PR 15%	*n* = 19 lesions
ECT	Matthiessen	2018	ECT	119	2 Months	CR: 50% PR: 21% SD: 18% PD: 8% Not evaluable: 3%	*n* = 90 patients
Topical therapy	Clive	1999	Miltefosine	25	18 Weeks	CR: 4% PR: 8% Minor response: 24% SD: 44% PD 20%	*n* = 25 patients
Topical therapy	Leonard	2001	Miltefosine	51	60 Days	CR: 8.3% PR: 25% SD: 7% PD: 16.7%	*n* = 24 patients
Topical therapy	Salazar	2017	Imiquimod	15	—	CR: 36% PR: 72%	*n* = 14 patients
Radiotherapy	Alizedah	2018	Surgery, CT, RT	1	3 Years	—	—
Radiotherapy	Lai	2003	As203 Gel, RT	7	—	CR: 42.9% PR: 42.9% SD: 14.3%	*n* = 7 patients
Aromatase inhibitors	Damaskos	2016	CT, AI	1	5 Years	—	—
Aromatase inhibitors	Ozet	2003	CT, RT, AI	1	4 Years	—	—
PDT	Allison	2001	PDT	9	6 Months	CR: 89% PR: 8% NR: 3%	*n*= 102 lesions
PDT	Lapes	1996	PDT	9	12 Months	CR: 33.3% PR: 22.2% NR: 22.2%	*n*= 7 patients
Targeted therapy	Blagden	2014	Class I PI3K inhibitor	38	—	—	—
Targeted therapy	Gui	2018	Bevacizumab	1	46 mo	—	—

AI, aromatase inhibitors; CR, complete response; CT, chemotherapy; ECT, electrochemotherapy; NR no response; PDT, photodynamic therapy, PR, partial response; RT radiotherapy.

Based on our clinical experience and the available evidence, we propose a personalized treatment model that integrates systemic and locoregional therapies. This approach aims to optimize therapeutic efficacy by tailoring interventions to the biological characteristics of the disease, the extent of cutaneous involvement, and the overall clinical condition of each patient with breast cancer cutaneous metastases. To facilitate clinical decision-making, we developed a practical flowchart that summarizes our proposed strategy and supports individualized treatment planning. This approach aims to maximize therapeutic efficacy and improve patient outcomes. Effective management of patients with cutaneous metastases from breast cancer should begin with a thorough and comprehensive evaluation of both the disease status and the patient’s overall condition, including an assessment of quality of life and symptoms. This process involves a global assessment with specific investigations to detect any distant metastases, using PET scan, CT scan, and MRI. Additionally, it is crucial to assess patient’s performance status using standardized scales, such as the Eastern Cooperative Oncology Group (ECOG) Performance Status, and to evaluate how the cutaneous metastases impact patient’s quality of life.

Based on the results of these evaluations, patients may be classified into one of the following three therapeutic scenarios:

1. Patients with resectable cutaneous metastases and no other sites of distant metastases: in this case, the patient should undergo a local excision of the cutaneous metastases even for recharacterizing receptor status and decide on the best systemic treatment. According to ESMO guidelines for oligometastatic disease, loco-regional treatments can be combined with systemic treatments after multidisciplinary discussion.^33^
2. Patients with non-resectable cutaneous metastases and/or cutaneous metastases associated with distant metastatic disease: For these patients, international guidelines for systemic treatment apply. If a good response is achieved for both distant and cutaneous metastases, the recommendation is to continue systemic therapy and integrate it with loco-regional treatment if a complete local response is not achieved. If there is a good response to distant metastases but a poor response to cutaneous metastases, systemic treatment should continue, and the most appropriate loco-regional treatment should be added, based on the disease characteristics and the patient’s condition. In cases of poor response to systemic therapy for distant metastases, it is suggested to modify the systemic treatment, if possible. If a systemic response is achieved after this change, the approaches described for favorable responses should be re-applied. In cases of systemic progression with no further treatment options available, low-impact loco-regional treatments, such as ECT or laser therapy, should be considered to manage symptoms (pain, bleeding, infections) and improve patient’s quality of life.
3. Patients with poor performance status, advanced age, and widespread cutaneous disease (with or without visceral metastases) who are not eligible for systemic treatments^109^: loco-regional treatment may still be indicated with a palliative intent to control local symptoms and complications related to cutaneous metastases.

This therapeutic approach is summarized in the flowchart we have developed for the treatment of cutaneous metastases from breast cancer (Figure 3).

This flowchart has been designed to guide clinicians through a structured decision-making process. The first step involves determining the extent of disease and the patient’s overall condition, which are essential for defining the intent of treatment. Multidisciplinary team discussion is strongly recommended at this stage, as it allows oncologists, surgeons, radiotherapists, and dermatologists to tailor the therapeutic plan based on receptor status, systemic disease burden, and patient preferences.

The flowchart emphasizes that treatment strategies should be individualized: patients with limited, resectable cutaneous lesions benefit from surgical excision and receptor reassessment, whereas patients with extensive or unresectable lesions require systemic approaches aligned with international guidelines. Loco-regional treatments, including ECT, laser therapy, or radiotherapy, are integrated at different steps depending on response patterns and symptom control needs. Finally, for frail patients with poor performance status, the algorithm prioritizes quality of life, focusing on symptom relief and minimizing treatment burden. This structured approach ensures a clear, evidence-based pathway for optimizing patient outcomes.

## Conclusion

Cutaneous metastases represent a significant challenge in the management of stage IV breast cancer, due to their clinical and biological complexity.

Breast cancer represents the most frequent cause of cutaneous metastases, highlighting the importance of a standardized care pathway for these patients. Although guidelines do not clearly distinguish between visceral and cutaneous metastases, local treatment options can be considered with the aim of improving local disease control, reducing symptoms from cutaneous metastases, and complementing systemic therapy in cases of well-controlled visceral disease.

Loco-regional therapies, such as ECT, radiotherapy, surgical excision, and, to a lesser extent, PDT, ILT, and topical therapies, offer targeted solutions for controlling localized disease and improving patient’s quality of life. The combination of these therapies with systemic treatments has been shown to increase therapeutic efficacy, as evidenced by several clinical studies. The choice of the most appropriate loco-regional therapy depends on various factors, including the number, size, and anatomical location of skin lesions, as well as patient’s overall clinical conditions.

In this review, we present a comprehensive and patient-tailored therapeutic model for managing cutaneous metastases arising from breast cancer, which integrates systemic therapies with loco-regional treatment options. The proposed strategy, summarized in our flowchart, is intended to serve as a practical guide to clinical decision-making, aiming to combine the local disease control achieved through loco-regional interventions with the survival benefits and broader therapeutic effects provided by systemic treatments.

In summary, the management of breast cancer-related cutaneous metastases demands an individualized and multidisciplinary approach. Regular discussions within a specialized team and ongoing reassessment of the patient’s clinical status are fundamental to determining the most appropriate timing for introducing loco-regional therapies in order to maximize symptom control and preserve quality of life. While recent advances in both systemic and loco-regional modalities have contributed to better outcomes, additional studies are required to refine treatment combinations and further improve long-term clinical results.

## Declarations

**Ethics approval and consent to participate:** Data from the patients included in this work were collected as part of a study approved by the Institutional Review Board (Istituto Oncologico Veneto I.O.V. (IRCCS), Padova) and carried out in accordance with the principles of the Declaration of Helsinki.

**Consent for publication:** Written informed consent for publication was obtained from patients involved in the study, and this was documented in their medical records.

**Availability of data and materials:** The datasets generated and analyzed during the current study are not publicly available due to our institution’s policies and the need to protect study participants’ privacy; however, they are partially available from the corresponding author upon reasonable request.

**ORCID iDs:** Paolo Del Fiore  https://orcid.org/0000-0002-2862-8014 Massimo Ferrucci  https://orcid.org/0009-0006-4635-333X

## References

[1] Rosen T. Cutaneous metastases. Med Clin North Am. 1980; 64(5): 885–900. 7432046 10.1016/s0025-7125(16)31572-3

[2] Siegel R Naishadham D Jemal A. Cancer statistics, 2013. CA Cancer J Clin 2013; 63(1): 11–30. 23335087 10.3322/caac.21166

[3] Teyateeti P Ungtrakul T. Retrospective review of cutaneous metastasis among 11,418 patients with solid malignancy: a tertiary cancer center experience. Medicine (Baltimore) 2021; 100(29): e26737. 10.1097/MD.0000000000026737 PMC8294925 34398051

[4] Moriarty JM Xing M Loh CT. Particle embolization to control life-threatening hemorrhage from a fungating locally advanced breast carcinoma: a case report. J Med Case Rep 2012; 6: 186. 22762410 10.1186/1752-1947-6-186 PMC3423047

[5] Reingold RE Corbett BE Blank NR , et al. Quality of life before and after treatment of cutaneous metastases with palliative radiotherapy. J Am Acad Dermatol 2022; 87(4): 868–870. 34826539 10.1016/j.jaad.2021.11.026 PMC9124716

[6] Queirós CS Filipe PL Cutaneous metastases from solid neoplasms in the 21st century: a retrospective study from a Portuguese tertiary care center. J Eur Acad Dermatol Venereol 2020; 34(6): 1218–1224. 31788857 10.1111/jdv.16120

[7] Krathen RA Orengo IF Rosen T. Cutaneous metastasis: a meta-analysis of data. South Med J 2003; 96(2): 164–167. 12630642 10.1097/01.SMJ.0000053676.73249.E5

[8] Gan EY Chio MT Tan WP. A retrospective review of cutaneous metastases at the National Skin Centre Singapore. Australas J Dermatol 2015; 56(1): 1–6. 10.1111/ajd.12194 25178874

[9] Ferrucci M Milardi F Passeri D , et al. Quality-of-life and oncological outcomes in male breast cancer: insights from an extensive 20-year experience. Cancers (Basel) 2025; 17(5): 829. 40075676 10.3390/cancers17050829 PMC11899275

[10] Caswell-Jin JL Sun LP Munoz D , et al. Analisi della mortalità per cancro al seno negli Stati Uniti-1975-2019. JAMA 2024; 331(3): 233–241. 38227031 10.1001/jama.2023.25881 PMC10792466

[11] Ibragimova MK Tsyganov MM Kravtsova EA , et al. Organ-specificity of breast cancer metastasis. Int J Mol Sci 2023; 24(21): 15625. 37958607 10.3390/ijms242115625 PMC10650169

[12] Lourenço C Conceição F Jerónimo C , et al. Stress in metastatic breast cancer: to the bone and beyond. Cancers (Basel) 2022; 14(8): 1881. 35454788 10.3390/cancers14081881 PMC9028241

[13] Zielonke N Gini A Jansen EEL , et al; EU-TOPIA consortium. Evidence for reducing cancer-specific mortality due to screening for breast cancer in Europe: a systematic review. Eur J Cancer 2020; 127: 191–206. 31932175 10.1016/j.ejca.2019.12.010

[14] Arozullah AM Calhoun EA Wolf M , et al. The financial burden of cancer: estimates from a study of insured women with breast cancer. J Support Oncol 2004; 2(3): 271–278. 15328826

[15] De Giorgi V Grazzini M Alfaioli B , et al. Cutaneous manifestations of breast carcinoma. Dermatol Ther 2010; 23(6):581–589. 21054704 10.1111/j.1529-8019.2010.01365.x

[16] Nava G Greer K Patterson J , et al. Metastatic cutaneous breast carcinoma: A case report and review of the literature. Can J Plast Surg 2009; 17(1): 25–27. 20190910 10.1177/229255030901700105 PMC2705310

[17] Johnson C Friedmann DP Gade A , et al. Cutaneous manifestation as initial presentation of metastatic breast cancer: a systematic review. Cutis 2021; 107(3): E29–E36. 10.12788/cutis.0223 33956620

[18] Lookingbill DP Spangler N Helm KF. Cutaneous metastases in patients with metastatic carcinoma: a retrospective study of 4020 patients. J Am Acad Dermatol 1993; 29(2 Pt 1): 228–236. 8335743 10.1016/0190-9622(93)70173-q

[19] Hu SC Chen GS Lu YW , et al. Cutaneous metastases from different internal malignancies: a clinical and prognostic appraisal. J Eur Acad Dermatol Venereol 2008; 22(6): 735–740. 18312322 10.1111/j.1468-3083.2008.02590.x

[20] Mordenti C Peris K Concetta Fargnoli M , et al. Cutaneous metastatic breast carcinoma. Acta Dermatovenerol 2000; 9(4): 143–148.

[21] Marneros AG Blanco F Husain S , et al. Classification of cutaneous intravascular breast cancer metastases based on immunolabeling for blood and lymph vessels. J Am Acad Dermatol 2009; 60(4): 633–638. 19293011 10.1016/j.jaad.2008.11.008

[22] Miguel TS Costa DAD Almeida APM , et al. Erysipelatoid carcinoma. Rev Assoc Med Bras (1992) 2018; 64(6): 492–497. 30304305 10.1590/1806-9282.64.06.492

[23] Alcaraz I Cerroni L Rütten A , et al. Cutaneous metastases from internal malignancies: a clinicopathologic and immunohistochemical review. Am J Dermatopathol 2012; 34(4): 347–393. 22617133 10.1097/DAD.0b013e31823069cf

[24] González-Martínez S Pizarro D Pérez-Mies B , et al. Clinical, pathological, and molecular features of breast carcinoma cutaneous metastasis. Cancers (Basel) 2021; 13(21): 5416. 34771579 10.3390/cancers13215416 PMC8582578

[25] Mayer JE Maurer MA Nguyen HT. Diffuse cutaneous breast cancer metastases resembling subcutaneous nodules with no surface changes. Cutis 2018; 101(3): 219–223. 29718016

[26] Lefebvre C Bachelot T Filleron T , et al. Mutational profile of metastatic breast cancers: a retrospective analysis. PLoS Med 2016; 13: e1002201. 10.1371/journal.pmed.1002201 PMC5189935 28027327

[27] Yates LR Knappskog S Wedge D , et al. Genomic evolution of breast cancer metastasis and relapse. Cancer Cell 2017; 32(2): 169–184.e7. 10.1016/j.ccell.2017.07.005 PMC5559645 28810143

[28] Rinaldi J Sokol ES Hartmaier RJ , et al. The genomic landscape of metastatic breast cancer: insights from 11,000 tumors. PLoS One 2020; 15(5): e0231999. 10.1371/journal.pone.0231999 PMC7202592 32374727

[29] González-Martínez S Pizarro D Pérez-Mies B , et al. Differences in the molecular profile between primary breast carcinomas and their cutaneous metastases. Cancers (Basel) 2022; 14(5): 1151. 35267459 10.3390/cancers14051151 PMC8909188

[30] Hussein MR. Skin metastasis: a pathologist’s perspective. J Cutan Pathol 2010; 37(9): e1–e20. 10.1111/j.1600-0560.2009.01469.x 19922483

[31] Pipkin CA Lio PA. Cutaneous manifestations of internal malignancies: an overview. Dermatol Clin 2008; 26(1): 1–15, vii. 18023767 10.1016/j.det.2007.08.002

[32] Tozbikian GH Zynger DL. A combination of GATA3 and SOX10 is useful for the diagnosis of metastatic triple-negative breast cancer. Hum Pathol 2019; 85: 221–227. 30468800 10.1016/j.humpath.2018.11.005

[33] Gennari A André F Barrios CH , et al. ESMO Clinical Practice Guideline for the diagnosis, staging and treatment of patients with metastatic breast cancer. Ann Oncol 2021; 32(12): 1475–1495. 34678411 10.1016/j.annonc.2021.09.019

[34] Dieci MV Barbieri E Piacentini F , et al. Discordance in receptor status between primary and recurrent breast cancer has a prognostic impact: a single-institution analysis. Ann Oncol 2013; 24(1): 101–108. 23002281 10.1093/annonc/mds248

[35] Grinda T Joyon N Lusque A , et al. Phenotypic discordance between primary and metastatic breast cancer in the large-scale real-life multicenter French ESME cohort. NPJ Breast Cancer 2021; 7(1): 41 33863896 10.1038/s41523-021-00252-6 PMC8052407

[36] Miglietta F Griguolo G Bottosso M , et al. Evolution of HER2-low expression from primary to recurrent breast cancer [published correction appears in *NPJ Breast Cancer*. 2021 Nov 24; 7(1): 149. DOI: 10.1038/s41523-021-00359-w.]. NPJ Breast Cancer 2021; 7(1): 137. 34642348 PMC8511010

[37] Boman C Zerdes I Mårtensson K , et al. Discordance of PD-L1 status between primary and metastatic breast cancer: a systematic review and meta-analysis. Cancer Treat Rev 2021; 99: 102257. 34237488 10.1016/j.ctrv.2021.102257

[38] Bottosso M Mosele F Michiels S , et al. Moving toward precision medicine to predict drug sensitivity in patients with metastatic breast cancer. ESMO Open 2024; 9(3): 102247. 38401248 10.1016/j.esmoop.2024.102247 PMC10982863

[39] Miglietta F Bottosso M Griguolo G , et al. Major advancements in metastatic breast cancer treatment: when expanding options means prolonging survival. ESMO Open 2022; 7(2): 100409. doi: 10.1016/j.esmoop.2022.100409. Erratum in: *ESMO Open* 2022; 7(3): 100472. 35227965 PMC8886005

[40] Baumann CK Castiglione-Gertsch M. Clinical use of selective estrogen receptor modulators and down regulators with the main focus on breast cancer. Minerva Ginecol 2009; 61(6): 517–539. 19942839

[41] Nabholtz J-MA . Long-term safety of aromatase inhibitors in the treatment of breast cancer. Ther Clin Risk Manag 2008; 4(1): 189–204. 18728707 10.2147/tcrm.s1566 PMC2503653

[42] Lee CI Goodwin A Wilcken N . Fulvestrant for hormone-sensitive metastatic breast cancer. Cochrane Database Syst Rev 20173; 1(1): CD011093. 10.1002/14651858.CD011093.pub2 PMC6464820 28043088

[43] Arshad M Paul AJ Kumar R. Skin metastasis from solid tumors: is targeted therapy making an impact? Cureus 2024; 16(11): e74362. 10.7759/cureus.74362 PMC11669381 39723293

[44] Droubi S Aqsa A Rehan M , et al. Rapid response of breast cancer cutaneous metastasis to single-agent palbociclib: a case report. Chemotherapy : 1–3. Epub ahead of print February 2021. DOI: 10.1159/000512499 33540405

[45] Bidard FC Kaklamani VG Neven P , et al. Elacestrant (oral selective estrogen receptor degrader) versus standard endocrine therapy for estrogen receptor-positive, human epidermal growth factor receptor 2-negative advanced breast cancer: results from the Randomized phase III EMERALD trial [published correction appears in J Clin Oncol. 2023 Aug 10;41(23):3962. doi: 10.1200/JCO.23.01239]. J Clin Oncol 2022; 40(28): 3246–3256. 35584336 PMC9553388

[46] Turner NC Oliveira M Howell SJ , et al. Capivasertib in hormone receptor-positive advanced breast cancer. N Engl J Med 2023; 388(22): 2058–2070. 37256976 10.1056/NEJMoa2214131 PMC11335038

[47] Modi S Jacot W Yamashita T , et al. Trastuzumab deruxtecan in previously treated HER2-low advanced breast cancer. N Engl J Med 2022; 387(1): 9–20. 35665782 10.1056/NEJMoa2203690 PMC10561652

[48] Rugo HS Bardia A Marmé F , et al. Sacituzumab govitecan in hormone receptor-positive/human epidermal growth factor receptor 2-negative metastatic breast cancer. J Clin Oncol 2022; 40(29): 3365–3376. 36027558 10.1200/JCO.22.01002

[49] Swain SM Baselga J Kim SB , et al. Pertuzumab, trastuzumab, and docetaxel in HER2-positive metastatic breast cancer. N Engl J Med 2015; 372(8): 724–734. 25693012 10.1056/NEJMoa1413513 PMC5584549

[50] Cortés J Kim SB Chung WP , et al. Trastuzumab deruxtecan versus trastuzumab emtansine for breast cancer. N Engl J Med 2022; 386(12): 1143–1154. 35320644 10.1056/NEJMoa2115022

[51] Giarratano T Miglietta F Giorgi CA , et al. Exceptional and durable responses to TDM-1 after trastuzumab failure for breast cancer skin metastases: potential implications of an immunological sanctuary. Front Oncol 2018; 8: 581. 30560092 10.3389/fonc.2018.00581 PMC6287048

[52] Conlin AK Chun BM Borges VF , et al. Cutaneous responses in HER2+ metastatic breast cancer: a retrospective case series of a phase 1b study of Tucatinib, an Oral HER2-specific inhibitor in combination with capecitabine and/or trastuzumab in third-line or later treatment, current problems in cancer: case reports. Curr Probl CancerCase Rep 2022; 7: 100170

[53] Morrison DG Marathe O. Breast cancer patient with widespread metastases and en Cuirasse presentation refractory to multiple lines of chemotherapy alone or with anti-HER-2-neu agents and radiation cleared rapidly and durably of all skin metastases by intravenous trastuzumab-deruxtecan. Oncol Case Report J 2022; 5(1): 1047.

[54] Cortes J Rugo HS Cescon DW , et al.; KEYNOTE-355 Investigators. Pembrolizumab plus chemotherapy in advanced triple-negative breast cancer. N Engl J Med 2022; 387(3): 217–226. 35857659 10.1056/NEJMoa2202809

[55] Schmid P Adams S Rugo HS , et al.; IMpassion130 Trial Investigators. Atezolizumab and nab-paclitaxel in advanced triple-negative breast cancer. N Engl J Med 2018; 379(22): 2108–2121. 30345906 10.1056/NEJMoa1809615

[56] Robson M Im SA Senkus E , et al. Olaparib for metastatic breast cancer in patients with a germline BRCA mutation [published correction appears in N Engl J Med. 2017 Oct 26;377(17):1700. doi: 10.1056/NEJMx170012.]. N Engl J Med 2017; 377(6): 523–533. 28578601

[57] Litton JK Hurvitz SA Mina LA , et al. Talazoparib versus chemotherapy in patients with germline BRCA1/2-mutated HER2-negative advanced breast cancer: final overall survival results from the EMBRACA trial. Ann Oncol 2020; 31(11): 1526–1535. 32828825 10.1016/j.annonc.2020.08.2098 PMC10649377

[58] Bardia A Hurvitz SA Tolaney SM , et al.; ASCENT Clinical Trial Investigators. Sacituzumab govitecan in metastatic triple-negative breast cancer. N Engl J Med 2021; 384(16): 1529–1541. 33882206 10.1056/NEJMoa2028485

[59] Di Menna G . Attività di sacituzumab govitecan nelle metastasi cutanee: descrizione di un caso [Activity of sacituzumab govitecan in skin metastases: description of a case.]. Recenti Prog Med 2024; 115(11): 90e–93e. 10.1701/4365.43612 39550673

[60] Spratt DE Gordon Spratt EA Wu S , et al. Efficacy of skin-directed therapy for cutaneous metastases from advanced cancer: a meta-analysis. J Clin Oncol 2014; 32(28): 3144–3155. 25154827 10.1200/JCO.2014.55.4634 PMC4979225

[61] Bertino G Groselj A Campana LG , et al. Electrochemotherapy for the treatment of cutaneous squamous cell carcinoma: the INSPECT experience (2008–2020). Front Oncol 2022; 12: 951662. 36203425 10.3389/fonc.2022.951662 PMC9531998

[62] Gehl J Sersa G Matthiessen LW , et al. Updated standard operating procedures for electrochemotherapy of cutaneous tumours and skin metastases. Acta Oncol 2018; 57(7): 874–882. 29577784 10.1080/0284186X.2018.1454602

[63] Russano F Del Fiore P Di Prata C , et al. The role of electrochemotherapy in the cutaneous and subcutaneous metastases from breast cancer: analysis of predictive factors to treatment from an Italian cohort of patients. Front Oncol 2021; 11: 772144. 34993137 10.3389/fonc.2021.772144 PMC8724516

[64] Cemazar M Sersa G. Recent advances in electrochemotherapy. Bioelectricity 2019; 1(4): 204–213. 34471824 10.1089/bioe.2019.0028 PMC8370294

[65] Cemazar M Milacic R Miklavcic D , et al. Intratumoral cisplatin administration in electrochemotherapy: antitumor effectiveness, sequence dependence and platinum content. Anticancer Drugs 1998; 9(6): 525–530. 9877240 10.1097/00001813-199807000-00002

[66] Campana LG Miklavčič D Bertino G , et al. Electrochemotherapy of superficial tumors—current status: basic principles, operating procedures, shared indications, and emerging applications. Semin Oncol 2019; 46(2): 173–191. 31122761 10.1053/j.seminoncol.2019.04.002

[67] Tsoneva I Semkova S Bakalova R , et al. Electroporation, electrochemotherapy and electro-assisted drug delivery in cancer. A state-of-the-art review. Biophys Chem 2022; 286: 106819. 35605496 10.1016/j.bpc.2022.106819

[68] Campana LG Marconato R Sieni E , et al. Elettrochemioterapia: meccanismo d'azione e risultati del trattamento locoregionale nei pazienti con tumori cutanei e metastasi superficiali [Electrochemotherapy: mechanism of action and clinical results in the locoregional treatment of patients with skin cancers and superficial metastases]. Recenti Prog Med 2016; 107(8): 422–433. [Italian.] 27571558 10.1701/2332.25066

[69] Bonferoni MC Rassu G Gavini E , et al. Electrochemotherapy of deep-seated tumors: state of art and perspectives as possible “EPR effect enhancer” to improve cancer nanomedicine efficacy. Cancers (Basel) 2021; 13(17): 4437. 34503247 10.3390/cancers13174437 PMC8431574

[70] Ursic K Kos S Kamensek U , et al. Potentiation of electrochemotherapy effectiveness by immunostimulation with IL-12 gene electrotransfer in mice is dependent on tumor immune status. J Control Release 2021; 332: 623–635. 33705828 10.1016/j.jconrel.2021.03.009

[71] Russano F Brugnolo D Bisetto G , et al. Electrochemotherapy treatment in a patient with an extended basal cell carcinoma of the face: a case report. J Pers Med 2024; 14(9): 984. 39338238 10.3390/jpm14090984 PMC11432816

[72] Dougherty TJ Gomer CJ Henderson BW , et al. Photodynamic therapy. J Natl Cancer Inst 1998; 90(12): 889–905. 9637138 10.1093/jnci/90.12.889 PMC4592754

[73] Di Prata C Mascherini M Ross AM , et al. Efficacy of electrochemotherapy in breast cancer patients of different receptor status: the INSPECT Experience. Cancers (Basel) 2023; 15(12): 3116. 37370726 10.3390/cancers15123116 PMC10295899

[74] Agostinis P Berg K Cengel KA , et al. Photodynamic therapy of cancer: an update. CA Cancer J Clin 2011; 61(4): 250–281. 21617154 10.3322/caac.20114 PMC3209659

[75] Jia J Wu X Long G , et al. Revolutionizing cancer treatment: nanotechnology-enabled photodynamic therapy and immunotherapy with advanced photosensitizers. Front Immunol 2023; 14: 1219785. 37860012 10.3389/fimmu.2023.1219785 PMC10582717

[76] Raszewski Ł Chyrek AJ Marciniak M , et al. High-dose-rate surface brachytherapy as a treatment option for renal cell carcinoma cutaneous metastases. J Contemp Brachytherapy 2021; 13(3): 331–337. 34122574 10.5114/jcb.2021.105947 PMC8170516

[77] Jacobson G Kaidar-Person O Haisraely O , et al. Palliative radiation therapy for symptomatic advance breast cancer. Sci Rep 2021; 11(1): 5282. 33674709 10.1038/s41598-021-84872-9 PMC7970854

[78] Vempati P Knoll MA Dharmarajan K , et al. Palliation of ulcerative breast lesions with radiation. Anticancer Res 2016; 36(9): 4701–4705. 27630316 10.21873/anticanres.11024

[79] Jacomina LE Swanson DM Mitchell MP , et al. Outcomes after palliative radiation therapy in patients with symptomatic locoregionally advanced breast cancer. Int J Radiat Oncol Biol Phys 2025; 122(2): 278–291. 39549757 10.1016/j.ijrobp.2024.11.065 PMC12172089

[80] Chatterjee S Chakrabarty S Santosham R , et al. Alleviating morbidity from locally advanced breast cancer using a practical and short radiation therapy regimen: results of the HYPORT Palliative Studies. Int J Radiat Oncol Biol Phys 2023; 116(5): 1033–1042. 36868522 10.1016/j.ijrobp.2023.02.008

[81] Webb K Gothard L Mohammed K , et al. Locoregional control and toxicity following high-dose hypofractionated and accelerated palliative radiotherapy regimens in breast cancer. Clin Oncol (R Coll Radiol) 2023; 35(9): e469–e477. 10.1016/j.clon.2023.06.006 37422360

[82] Iyer P. Locally advanced breast cancer: a systemic disease with a locoregional symptom burden. Is it time to challenge the “no local treatment” stereotype? Int J Radiat Oncol Biol Phys 2025; 122(2): 292–293. 40382165 10.1016/j.ijrobp.2025.03.008

[83] Hoeltgen L Meixner E Hoegen P , et al. Palliative radiotherapy for symptomatic locally advanced breast cancer. Technol Cancer Res Treat 2023; 22: 15330338231164537. 10.1177/15330338231164537 PMC10103240 37038619

[84] Bhave P Hong A Lo SN , et al. Efficacy and toxicity of adjuvant radiotherapy in recurrent melanoma after adjuvant immunotherapy. J Immunother Cancer 2023; 11(3): e006629. 10.1136/jitc-2022-006629 PMC10008434 36889810

[85] Merino T Tran WT Czarnota GJ. Re-irradiation for locally recurrent refractory breast cancer. Oncotarget 2015; 6(33): 35051–35062. 26459388 10.18632/oncotarget.6036 PMC4741508

[86] Ferrucci M Milardi F Passeri D , et al. Pilot study: blue light photobiomodulation for the treatment of complicated wounds in breast surgery. Plast Reconstr Surg Glob Open 2025; 13(7): e6989. 10.1097/GOX.0000000000006989 PMC12303515 40727624

[87] Lin YL. Reirradiation of recurrent breast cancer with proton beam therapy: a case report and literature review. World J Clin Oncol 2019; 10(7): 256–268. 31396475 10.5306/wjco.v10.i7.256 PMC6682500

[88] Matthiessen LW Keshtgar M Curatolo P , et al. Electrochemotherapy for breast cancer-results from the INSPECT Database. Clin Breast Cancer 2018; 18(5): e909–e917. 10.1016/j.clbc.2018.03.007 29673795

[89] Bliley R Avant A Medina TM , et al. Radiation and melanoma: where are we now? Curr Oncol Rep 2024; 26(8): 904–914. 38822928 10.1007/s11912-024-01557-y

[90] Fritz P Hensley FW Berns C , et al. Long-term results of pulsed irradiation of skin metastases from breast cancer. Effectiveness and sequelae. Strahlenther Onkol 2000; 176(8): 368–376. 10987020 10.1007/pl00002345

[91] Storm FK Sparks FC Morton DL. Treatment for melanoma of the lower extremity with intralesional injection of bacille Calmette Guérin and hyperthermic perfusion. Surg Gynecol Obstet 1979; 149(1): 17–21. 451822

[92] Byers BA Temple-Oberle CF Hurdle V , et al. Treatment of in-transit melanoma with intra-lesional interleukin-2: a systematic review. J Surg Oncol 2014; 110(6): 770–775. 24996052 10.1002/jso.23702

[93] Nadler A Look Hong NJ Alavi N , et al. Lesional therapies for in-transit melanoma. J Surg Oncol 2020; 122(6): 1050–1056. 32668038 10.1002/jso.26121

[94] Kumar A Taghi Khani A Sanchez Ortiz A , et al. GM-CSF: a double-edged sword in cancer immunotherapy. Front Immunol 2022; 13: 901277. 35865534 10.3389/fimmu.2022.901277 PMC9294178

[95] Yu J Wang Q Wang L , et al. PD-1 inhibitor combined with SBRT, GM-CSF, and thymosin alpha-1 in metastatic breast cancer: a case report and literature review. Medicine (Baltimore) 2024; 103(34): e39271. 10.1097/MD.0000000000039271 PMC11346870 39183403

[96] Eubank TD Roberts RD Khan M , et al. Granulocyte macrophage colony-stimulating factor inhibits breast cancer growth and metastasis by invoking an anti-angiogenic program in tumor-educated macrophages. Cancer Res 2009; 69(5): 2133–2140. 19223554 10.1158/0008-5472.CAN-08-1405 PMC2722508

[97] Anderson KS Erick TK Chen M , et al. The feasibility of using an autologous GM-CSF-secreting breast cancer vaccine to induce immunity in patients with stage II-III and metastatic breast cancers. Breast Cancer Res Treat 2022; 194(1): 65–78. 35482127 10.1007/s10549-022-06562-y PMC9046531

[98] Green DS Bodman-Smith MD Dalgleish AG , et al. Phase I/II study of topical imiquimod and intralesional interleukin-2 in the treatment of accessible metastases in malignant melanoma. Br J Dermatol 2007; 156(2): 337–345. 17223875 10.1111/j.1365-2133.2006.07664.x

[99] Florin V Desmedt E Vercambre-Darras S , et al. Topical treatment of cutaneous metastases of malignant melanoma using combined imiquimod and 5-fluorouracil. Invest New Drugs 2012; 30(4): 1641–1645. 21748297 10.1007/s10637-011-9717-2

[100] Jouret G Gonne E Quatresooz P , et al. Cutaneous breast cancer metastases successfully treated using an oxygen flow assisted topical administration of methotrexate (OFAMTX). Dermatol Ther (Heidelb) 2020; 10(4): 855–861. 32415574 10.1007/s13555-020-00393-9 PMC7367989

[101] Lebas E Chapelier C Quatresooz P , et al. Exploratory assessment of oxygen flow-assisted cutaneous administration of methotrexate for superficial basal cell carcinoma, mycosis fungoides, and extramammary paget disease. J Invest Dermatol 2020; 140(3): 583–592. 31513804 10.1016/j.jid.2019.08.443

[102] Lacouture ME Goldfarb SB Markova A , et al. Phase 1/2 study of topical submicron particle paclitaxel for cutaneous metastases of breast cancer. Breast Cancer Res Treat 2022; 194(1): 57–64. 35471470 10.1007/s10549-022-06584-6 PMC9167189

[103] Salazar LG Lu H Reichow JL , et al. Topical imiquimod plus nab-paclitaxel for breast cancer cutaneous metastases: a phase 2 clinical trial. JAMA Oncol 2017; 3(7): 969–973. 28114604 10.1001/jamaoncol.2016.6007 PMC5824239

[104] Clive S Gardiner J Leonard RC. Miltefosine as a topical treatment for cutaneous metastases in breast carcinoma. Cancer Chemother Pharmacol 1999; 44(Suppl.): S29–S30. 10.1007/s002800051114 10602908

[105] Strobbe LJ Nieweg OE Kroon BB. Carbon dioxide laser for cutaneous melanoma metastases: indications and limitations. Eur J Surg Oncol 1997; 23(5): 435–438. 9393574 10.1016/s0748-7983(97)93726-4

[106] Salvadori B Rovini D Squicciarini P , et al. Surgery for local recurrences following deficient radical mastectomy for breast cancer: a selected series of 39 cases. Eur J Surg Oncol 1992; 18(5): 438–441. 1426293

[107] Huang S Parekh V Waisman J , et al. Cutaneous metastasectomy: is there a role in breast cancer? A systematic review and overview of current treatment modalities. J Surg Oncol 2022; 126(2): 217–238. 35389520 10.1002/jso.26870 PMC9545220

[108] Bastrup FA Vissing M Gehl J. Electrochemotherapy with intravenous bleomycin for patients with cutaneous malignancies, across tumour histology: a systematic review. Acta Oncol 2022; 61(9): 1093–1104. 36036195 10.1080/0284186X.2022.2110385

[109] Ferrucci M Passeri D Milardi F , et al. Surgery plays a leading role in breast cancer treatment for patients aged ⩾90 years: a large retrospective cohort study. Ann Surg Oncol 2024; 31(11): 7377–7391. 39098873 10.1245/s10434-024-15790-z PMC11452447

